# Hyperreflective Walls in Foveal Cystoid Spaces as a Biomarker of Diabetic Macular Edema Refractory to Anti-VEGF Treatment

**DOI:** 10.1038/s41598-020-64332-6

**Published:** 2020-04-29

**Authors:** Noriko Terada, Tomoaki Murakami, Akihito Uji, Yoko Dodo, Yuki Mori, Akitaka Tsujikawa

**Affiliations:** Department of Ophthalmology and Visual Sciences, Kyoto University Graduate School of Medicine, Kyoto, Japan

**Keywords:** Prognostic markers, Retinal diseases

## Abstract

Diabetic macular edema (DME) refractory to anti-VEGF drugs is a socioeconomic burden. In this retrospective study, we investigated the relationship between DME remission and *hyperreflective walls in foveal cystoid spaces*, a novel finding on spectral domain optical coherence tomography (SD-OCT) images in DME. In a cross-sectional study, we assessed the relationship between hyperreflective walls in foveal cystoid spaces and other OCT findings in 110 eyes of 110 DME patients. Hyperreflective walls were delineated in 27 of 171 foveal cystoid spaces. Eyes with hyperreflective walls in foveal cystoid spaces had poorer visual acuity and more severe photoreceptor disruption than did those without such findings (*P* = 0.008 and *P* < 0.001, respectively). In the other longitudinal study, we evaluated the relationship between this finding and the remission in 54 eyes of 51 DME patients treated with as-needed ranibizumab injections for 24 months. Foveal cystoid spaces with hyperreflective walls were often persistent, and the cumulative rates of DME remission among eyes with and without the hyperreflective walls were 7.7% (1 eye) and 48.8% (20 eyes) at 18 months (hazard ratio, 0.092; *P* = 0.025). We characterized *hyperreflective walls in foveal cystoid spaces* and designated them as a predictor of no DME remission under ranibizumab injections.

## Introduction

Diabetic macular edema (DME) is a major cause of visual dysfunction in diabetic patients^1^. The breakdown of the blood-retinal barrier (BRB) leads to the accumulation of intracellular and extracellular fluids in the retinal parenchyma^2^. Two major clinical examinations, fluorescein angiography (FA) and spectral domain optical coherence tomography (SD-OCT), delineate the various patterns of vascular hyperpermeability and neuroglial impairment^3^. However, how the diversity in clinical findings influences the responsiveness to individual interventions remains poorly understood.

Structural analyses using OCT improve the qualities of medical management for diabetic patients. The measurement of central subfield (CSF) thickness provides an objective and quantitative diagnosis of center-involved DME. There are modest correlations between CSF thickness and visual acuity (VA) in DME^4^. The qualitative assessment of retinal morphologies on sectional OCT images allows us to understand the pathological mechanisms more precisely^3,5^. Macular thickening is composed of cystoid macular edema (CME), serous retinal detachment (SRD), and sponge-like retinal swelling on OCT images^6^. CME is associated with enlarged foveal avascular zone and the patterns of fluorescein pooling^7,8^. In addition, structural OCT revealed a few findings in cystoid spaces, e.g., high reflectance, heterogeneous reflectivity, and hyperreflective foci^9,10^. Some OCT findings are biomarkers to predict the early response to anti-vascular endothelial growth factor (VEGF) treatment or steroids for DME^11–14^. Hyperreflective intraretinal spots and subfoveal neuroretinal detachment decrease after anti-VEGF therapy^15^. This idea suggests the clinical feasibility of OCT parameters, although whether they are predictors of the long-term responses for DME remains largely unknown.

Among several therapeutic strategies, anti-VEGF drugs are the first-line treatment for DME^16^. Despite its significant efficacy, we need to consider the severe socioeconomic burden and higher cost-per-quality-adjusted life years of anti-VEGF treatment than those of other conventional therapies^17^. Major clinical trials using pro re nata (PRN) dosing revealed that the median treatment frequency is reduced in the second year and beyond, although some cases need frequent injections at later visits^16,18^. We should consider several possible explanations for the refractory disease, e.g., tachyphylaxis to these drugs, molecular mechanisms other than VEGF, and retinal degeneration^19,20^. These explanations might support the decision for additional therapeutic strategies to some extent, although clinical fundus findings in the refractory DME should be elucidated.

In this study, we characterized *hyperreflective walls in foveal cystoid spaces*, a novel OCT finding, and investigated whether this finding can predict DME refractory to anti-VEGF drugs.

## Results

### Characteristics of hyperreflective walls in foveal cystoid spaces

**Figure 1:**
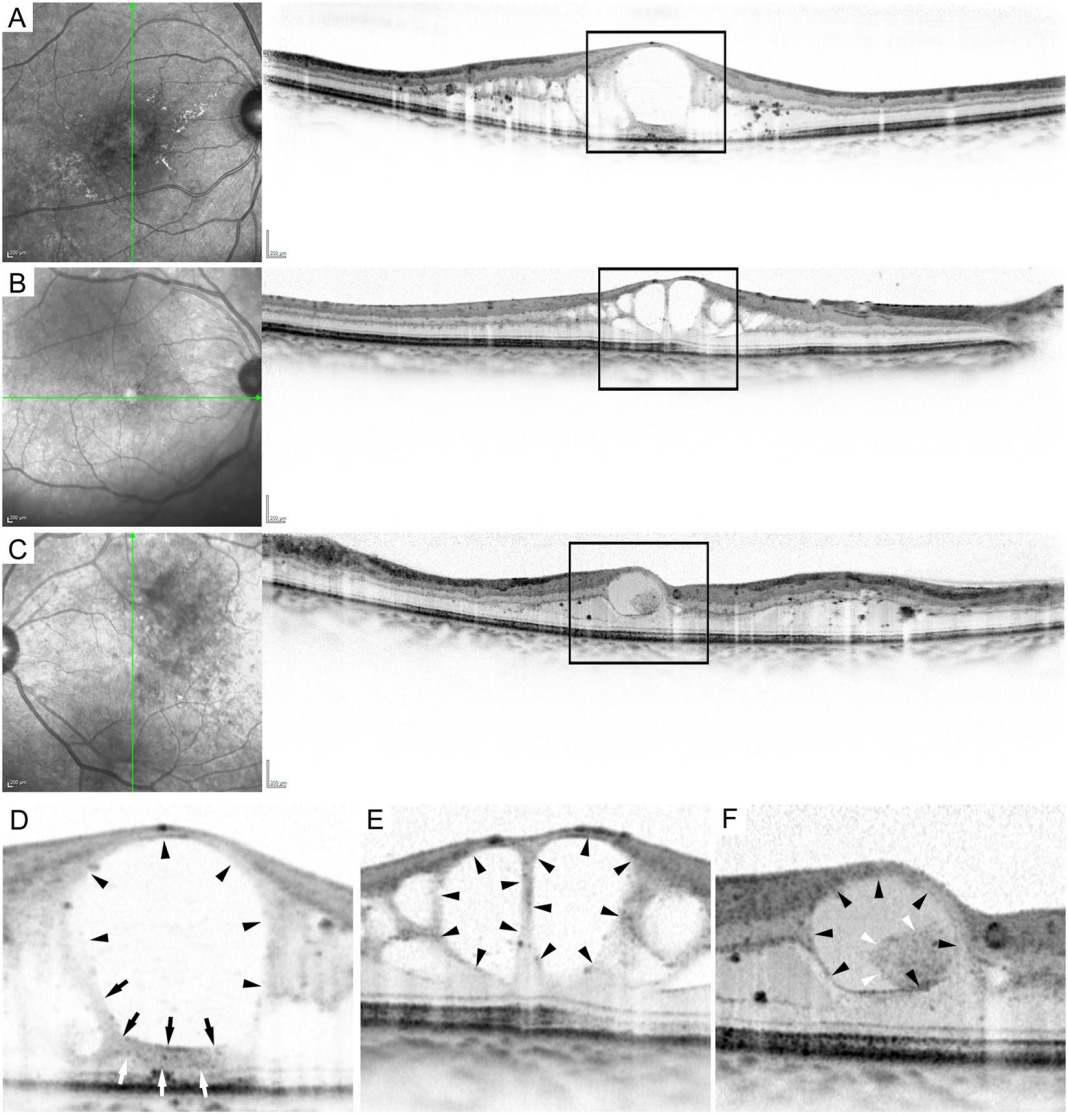
Hyperreflective walls in foveal cystoid spaces on sectional OCT images in DME. (**A,D**) Hyperreflective walls (white and black arrows) in the bottom of foveal cystoid spaces in a 57-year-old man with moderate NPDR. (**B,E**) Foveal cystoid spaces without hyperreflective walls in a 71-year-old woman with moderate NPDR. (**C,F**) Hyperreflective deposits (white arrowheads) within cystoid spaces in a 56-year-old man with severe NPDR. Black arrows and arrowheads = contour of cystoid spaces. (**D–F)** are magnified images of the rectangles in (**A–C)**, respectively. Scale bar = 200 μm.

**Figure 2:**
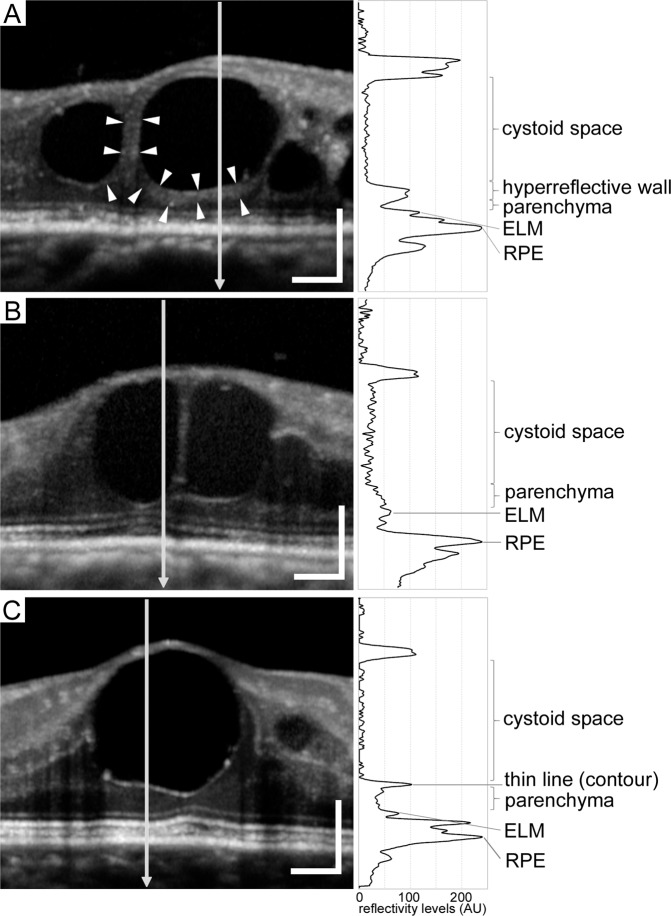
Reflective patterns of walls in foveal cystoid spaces in DME. Reflective profiles in foveal cystoid spaces show that walls of cystoid spaces are composed of thick and hyperreflective bands (**A**), retinal parenchyma (**B**), or thin and hyperreflective lines as a contour due to optical differences (**C**). Hyperreflective bands in panel A are defined as *hyperreflective walls in foveal cystoid spaces* (arrowheads). Right column = reflectivity levels along the arrows in the left column, which constructed using ‘Plot profile’ function in ImageJ version 1.52 software (NIH, Bethesda, MD). Scale bar = 200 μm.

We retrospectively reviewed 110 eyes of 110 patients with treatment-naïve center-involving DME. Patient characteristics are shown in Table S1. *Hyperreflective walls* were delineated in 27 (15.8%) of 171 foveal cystoid spaces (Figs. 1, 2). Only seven (3.6%) of 192 parafoveal cystoid spaces had the walls. This OCT finding was often delineated in the whole bottom and sometimes in the top or septa (or lateral boundaries) and their thicknesses were 51μm (48–56). The relative reflectivity of the hyperreflective walls was 51.8 (44.0–58.5). Cystoid spaces with the hyperreflective walls had lower levels of OCT reflectivity (*P* = 0.036) and greater width (*P* = 0.004) than did those without such walls. Hyperreflective foci were delineated in cystoid spaces with the hyperreflective walls more frequently than those without such walls (*P* = 0.044; Table 1).

**Table 1:** Association between hyperreflective walls and other OCT findings in individual 171 cystoid spaces in 110 eyes with foveal cystoid spaces.

	Hyperreflective walls in foveal cystoid spaces		*P*-value
	Present (n = 27 cystoid spaces)	Absent (n = 144 cystoid spaces)	
Height (μm)	293 (209–420)	252 (200–317)	0.089
Width (μm)	514 (405–739)	364 (280–511)	0.004
Reflectivity levels (AU)	10.8 (3.8–20.4)	14.8 (4.1–28.0)	0.036
Homogenous/heterogeneous	21/6	101/43	0.494
Hyperreflective foci (present/absent)	23/4	94/50	0.044

Eyes with hyperreflective walls in foveal cystoid spaces had poorer logarithm of the minimum angle of resolution (logMAR) VA than did those without such findings, although there were no differences in CSF thicknesses (Table 2). There was no association of the hyperreflective walls with subretinal fluid, vitreoretinal abnormalities, and hyperreflective foci in the inner and outer retinal layers (Table 2). In contrast, the disrupted ellipsoid zone of photoreceptors (EZ) line and disorganization of the retinal inner layers (DRIL) extent were longer in eyes with hyperreflective walls in foveal cystoid spaces than in those without such walls (*P* < 0.001 and *P* = 0.032, respectively).

**Table 2:** Comparisons of ocular parameters between eyes with and without hyperreflective walls in foveal cystoid spaces.

Parameter	Hyperreflective wall in foveal cystoid spaces		*P*-value
	Present (n = 24 eyes)	Absent (n = 86 eyes)	
LogMAR VA	0.427 (0.222–0.793)	0.155 (0.046–0.398)	0.008
International classification			
Mild NPDR	0	2	0.850
Moderate NPDR	12	41	
Severe NPDR	8	23	
PDR	4	20	
Pseudophakia	7	21	0.608
Panretinal photocoagulation	8	38	0.362
CSF thickness (μm)	500 (364–567)	430 (340–500)	0.145
Subretinal fluid	2 (8.3%)	19 (22.1%)	0.154
Vitreoretinal abnormalities	3 (12.5%)	12 (14.0%)	1.000
Disrupted EZ line (%)	20.7 (8.3–57.5)	0.0 (0.0–10.2)	<0.001
DRIL (μm)	521 (390–709)	406 (223–545)	0.032
Hyperreflective foci in the inner retinal layers	17 (70.8%)	70 (81.4%)	0.268
Hyperreflective foci in the outer retinal layers	12 (50.0%)	28 (32.6%)	0.151

### Course of hyperreflective walls in foveal cystoid spaces in eyes treated with anti-VEGF treatment

**Figure 3:**
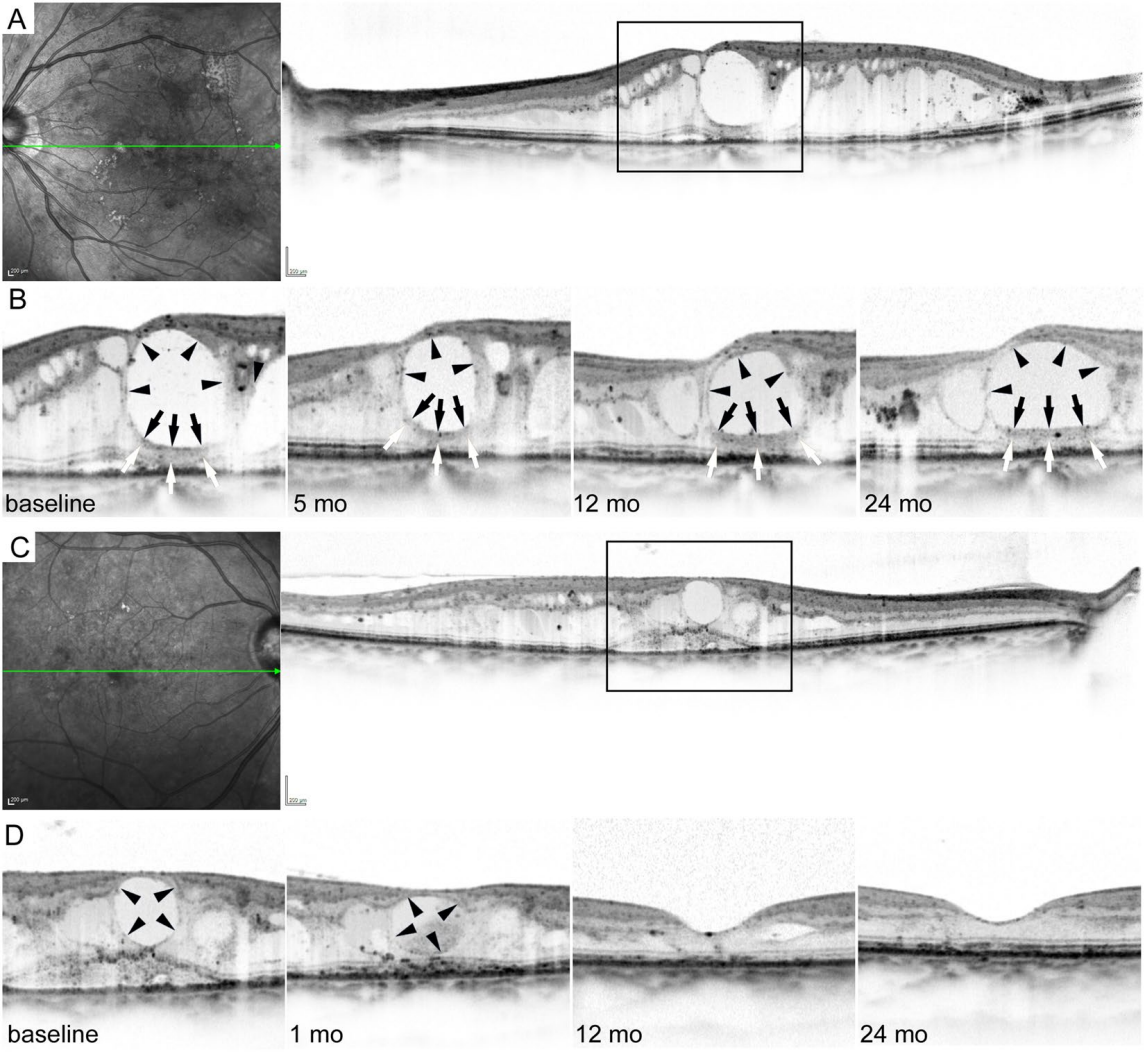
The courses of hyperreflective walls in foveal cystoid spaces under IVR PRN injections for DME. (**A**) The OCT image at baseline shows foveal cystoid spaces with hyperreflective walls in a 63-year-old woman with moderate NPDR. (**B**) The magnified images of the fovea (rectangle in panel A) at each time point. CSF thickness was decreased after ranibizumab injections, although foveal cystoid spaces with hyperreflective walls (white and black arrows) were persistent over two years despite 16 IVR injections. (**C**) The baseline OCT image delineates foveal cystoid spaces without hyperreflective walls in a 67-year-old woman with moderate NPDR. (**D**) The course of the magnified images (rectangle in panel C) shows the gradual reduction in retinal thicknesses and complete DME remission after 7 IVR injections. Black arrows and arrowheads = contour of foveal cystoid spaces. Scale bar = 200 μm.

**Figure 4:**
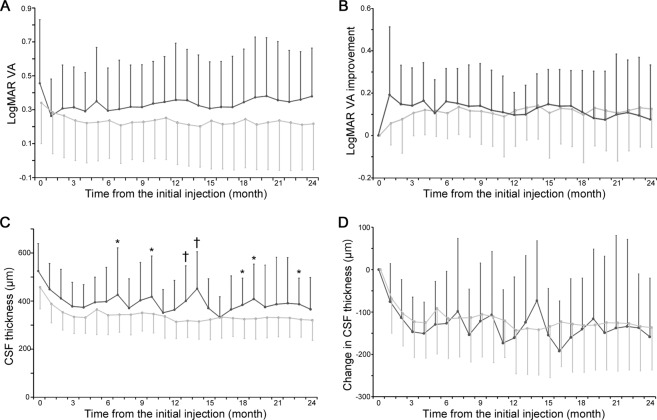
Efficacy of ranibizumab injections for DME in eyes with and without hyperreflective walls in foveal cystoid spaces. LogMAR VA (**A**) and its improvement (**B**) in 13 eyes with hyperreflective walls in foveal cystoid spaces (dark gray) and 41 eyes without such findings (light gray). (**C**) CSF thicknesses are greater in eyes with hyperreflective walls than in eyes without such findings at several time points. *P < 0.05; ^†^*P* < 0.01. (**D**) Changes in CSF thickness.

In the longitudinal study, we reviewed 54 eyes of 51 patients with center-involved DME after the exclusion of 75 eyes among 129 eyes that met the inclusion criteria (Table S2). Improvement of logMAR VA and decrease in CSF thickness were maintained until the 24-month visit (Figs. 3 and 4). Hyperreflective walls in foveal cystoid spaces, disrupted EZ line, and DRIL extent were decreased (Fig. S1).

**Figure 5:**
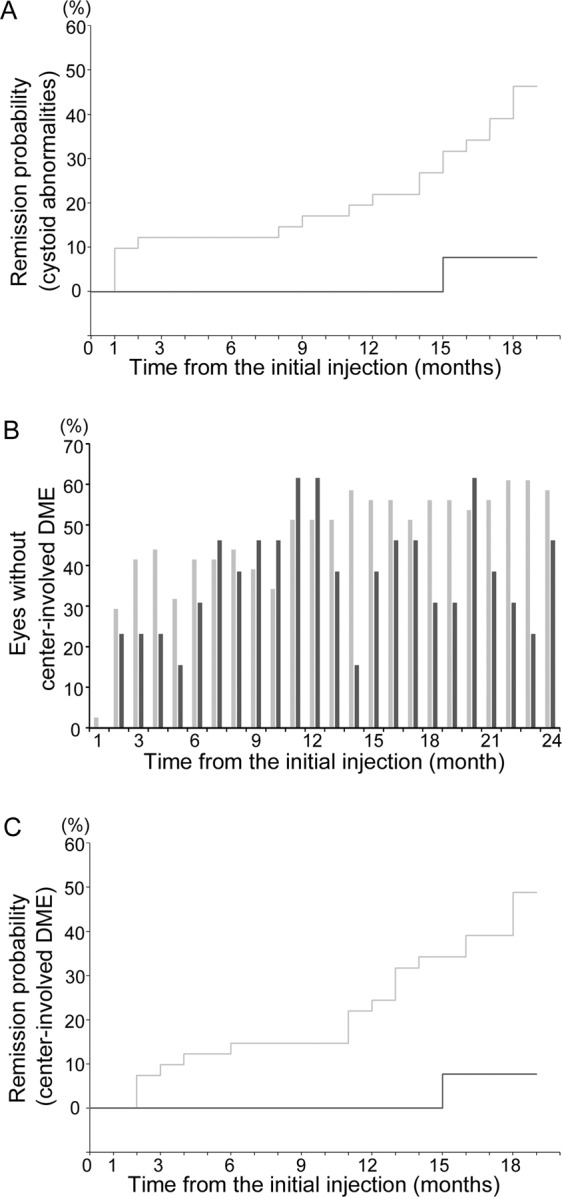
Cumulative ratios of DME remission under as-needed ranibizumab injections. (**A**) The cumulative ratios of the remission of cystoid abnormalities in eyes with hyperreflective walls were smaller than those in eyes without the walls. (**B**) The ratios of eyes without center-involved DME at individual time points. (**C**) The cumulative ratio of DME remission among two groups. The ratios were smaller in eyes with hyperreflective walls. dark gray = eyes with hyperreflective walls in foveal cystoid spaces, light gray = those without hyperreflective walls.

We evaluated the course of hyperreflective walls in foveal cystoid spaces in eyes treated with ranibizumab injections. All 54 eyes had cystoid abnormalities at baseline and gradually decreased to 20 eyes (37.0%) at 24 months. In contrast, eyes with hyperreflective walls in foveal cystoid spaces persisted from 13 eyes at baseline to 19 and 13 eyes at 12- and 24-month visits, respectively (Fig. 3). Among 13 eyes with hyperreflective walls at baseline, 13 (100%) and 9 (69.2%) eyes had cystoid abnormalities at 12 and 24 months, respectively. In 41 eyes without pretreatment hyperreflective walls, cystoid abnormalities were delineated in only 19 (46.3%) and 11 (26.8%) eyes at 12 and 24 months (*P* < 0.001 in both comparisons at 12 and 24 months). The ratios of remission of cystoid abnormalities among eyes with and without hyperreflective walls at baseline increased to 7.7% (1 eye) and 46.3% (19 eyes) at 18 months (*P* = 0.048; Fig. 5A).

### Hyperreflective walls in foveal cystoid spaces at baseline as a predictor of no DME remission until 18 months

There were no differences in VA improvement and the decrease in CSF thickness between eyes with and without the hyperreflective walls at baseline (Fig. 4B,D). LogMAR VA did not differ, whereas CSF thicknesses were greater in eyes with hyperreflective walls than in eyes without them at several time points (Fig. 4A,C). The ratios of eyes without center-involved DME were variable in eyes with the hyperreflective walls, compared to those without the walls (Fig. 5B). The cumulative rates of DME remission among eyes with and without the hyperreflective walls were 7.7% (1 eye) and 48.8% (20 eyes) at 18 months (Fig. 5C). According to multivariate analyses, the hyperreflective walls at baseline had significant higher risk of no remission until the 18-month visit (hazard ratio [HR], 0.092; 95% confidence interval [CI], 0.011–0.742; *P* = 0.025) (Table 3).

**Table 3:** Cox proportional hazards regression model for variables associated with DME remission until 18 months under as-needed ranibizumab injections.

Variables at baseline	Univariate		multivariate	
	HR (95% CI)	*P*-value	HR (95% CI)	*P*-value
Age (years)	1.007 (0.962–1.054)	0.768	—	—
Female	0.678 (0.263–1.750)	0.422	—	—
Hemoglobin A1c (%)	0.189 (0.017–2.083)	0.174	—	—
Systemic hypertension	0.849 (0.357–2.015)	0.710	—	—
logMAR VA	1.045 (0.223–4.893)	0.955	—	—
PDR	0.455 (0.106–1.953)	0.289	—	—
Pseudophakia	0.722 (0.311–1.913)	0.576	—	—
Panretinal photocoagulation	0.354 (0.149–0.844)	0.019	0.347 (0.135–0.889)	0.027
CSF thickness (μm)	0.997 (0.992–1.001)	0.147	—	—
Hyperreflective walls in foveal cystoid spaces	0.124 (0.017–0.925)	0.042	0.092 (0.011–0.742)	0.025
Hyperreflective foci in foveal cystoid spaces	0.563 (0.233–1.359)	0.201	—	—
Subretinal fluid	0.924 (0.215–3.968)	0.915	—	—
Vitreoretinal abnormalities	0.045 (0.000–80.190)	0.416	—	—
Disrupted EZ line (%)	1.001 (0.986–1.017)	0.882	1.018 (1.001–1.036)	0.043
DRIL (μm)	1.001 (0.985–1.018)	0.864	—	—
Hyperreflective foci in the inner retinal layers	0.506 (0.196–1.306)	0.159	—	—
Hyperreflective foci in the outer retinal layers	0.875 (0.372–2.062)	0.761	—	—

## Discussion

In the cross-sectional study, we defined hyperreflective walls in foveal cystoid spaces as a novel structural OCT finding in DME. This OCT finding was delineated in the top or bottom of cystoid spaces or the septa between the spaces. Hyperreflective foci were frequently accompanied by hyperreflective walls. Since hyperreflective foci generally correspond to lipid-laden macrophages, inflammatory responses might contribute to the development of the hyperreflective walls. In the longitudinal study, foveal cystoid spaces with hyperreflective walls were persistent, whereas eyes with cystoid abnormalities were decreased under anti-VEGF treatment. This finding is consistent with poorer VA and more severe EZ disruption in eyes with hyperreflective walls. In particular, statistical analyses revealed that the hyperreflective walls in foveal cystoid spaces at baseline predicted unremitted DME under PRN intravitreal ranibizumab (IVR) injections.

We could not determine what type of histological lesions correspond to the hyperreflective walls in foveal cystoid spaces in this study. Several histological publications demonstrated Henle’s fibers or Müller cells in the lateral walls and photoreceptor cells in the bottom walls^21^. A recent surgical publication documented that fibrinogen-rich cystoid spaces resected during vitrectomy are encapsulated by collagen fibrils in DME^22^. The clear boundaries of hyperreflective walls may allow us to speculate that collagenous capsules correspond to hyperreflective walls in cystoid spaces and contribute to refractoriness to anti-VEGF treatment. Another possible explanation is that cystoid spaces might be surrounded by gliotic tissues^23^. In this study, most cystoid spaces with hyperreflective walls were accompanied by hyperreflective foci. Hyperreflective foci may correspond to lipid-laden macrophages and suggest inflammatory responses^2,10^. These macrophages could promote fibrotic/gliotic scar formation of cystoid spaces^24^. The third possibility is fibrin deposition, as in the case of intraretinal and subretinal deposits in chorioretinal vascular diseases^22^. The hyperreflective walls at the bottom of cystoid spaces might correspond to the compressed retina layers, although we carefully confirmed the different reflectivity between the wall and nearby retinal parenchyma. Further investigation should elucidate the correspondence between histological and OCT findings.

Cystoid abnormalities sometimes relapsed in the second year, although most serous retinal detachment (SRD) was resolved until 12 months (Data not shown). Since SRD development is mediated via BRB breakdown, anti-VEGF drugs might decrease vascular hyperpermeability and subsequently resolve SRD^25,26^. In contrast, the pathophysiological mechanisms in foveal cystoid spaces are complicated; extravasation, ischemia, degeneration, vitreoretinal traction, intracytoplasmic swelling of Müller cells^7,21,27^. Anti-VEGF treatment can neither repair capillary nonperfusion nor neurodegeneration or do not remove vitreoretinal traction. This may partly explain the refractoriness of foveal cystoid spaces. Additionally, the current study designated hyperreflective walls in foveal cystoid spaces as a novel predictor of unremitted DME. The OCT reflectivity was lower in cystoid spaces with hyperreflective walls. It may allow us to speculate that the hyperreflective walls correspond to capsules of fibrotic tissues or fibrin deposits and maintain the structure of cystoid spaces even under anti-VEGF treatment. Another possibility is that some of cystoid spaces with hyperreflective walls correspond to cystoid macular degeneration as in the case of neovascular age-related macular degeneration, because such cystoid spaces sometimes had concave and straight border in this study^28,29^.

Frequent injections of expensive anti-VEGF drugs are the first-line treatment for DME.^17^ We speculate that remission induction is one of important factors to reduce a socioeconomic burden in this chronic disease. In this study, we designated hyperreflective walls in foveal cystoid spaces at baseline as a predictor of no DME remission until 18 months under PRN IVR injections. Since eyes without the hyperreflective walls often reached remission, they may be suitable for anti-VEGF treatment among several therapeutic strategies. In contrast, most eyes with hyperreflective walls in foveal cystoid spaces did not reach remission until 18 months, which might encourage us to consider the switch to or the combination with other interventions for such cases^30,31^. Future studies should elucidate whether other regimens, e.g., treat-and-extend or monthly regimens, of anti-VEGF injections allow eyes with the hyperreflective walls to achieve remission.

In the cross-sectional study, hyperreflective walls in foveal cystoid spaces were associated with visual impairment in DME. In the longitudinal study, VA did not differ in eyes with and without such OCT finding. The discrepancy might be dependent on the differences in participants. Although the former study included cases with good VA, some patients with good VA did not wish to receive anti-VEGF treatment and were not included into the longitudinal study. We may speculate that VA was not associated with the hyperreflective walls in DME cases with VA reduction.

There are several limitations in this retrospective and preliminary study with small sample sizes. In particular, the retrospective nature is a serious concern in predicting treatment responses in a longitudinal study. Since most patients were referred to our institute without enough information of DME duration, we could not evaluate the disease chronicity. The sectional OCT images were acquired and averaged using a specific SD-OCT machine and its equipped software. Whether this OCT finding is delineated on a single scan image using other OCT devices remains to be elucidated. Three-dimensional analyses would improve the localization of hyperreflective walls. Since several OCT findings were subjectively assessed in this study, future studies should develop procedures to evaluate them objectively. We selected the PRN dosing of IVR injections, although further studies have to confirm the generalizability to other regimens, other anti-VEGF drugs, or other interventions. All participants were Asian in a single institute; thus, we should plan to confirm the reproducibility in other races in prospective multicenter studies.

We documented *hyperreflective walls in foveal cystoid spaces* as a novel OCT finding and characterized its clinical relevance in DME. This OCT finding may be designated as a predictor of no DME remission under as-needed ranibizumab injections.

## Methods

### Patients

We prepared two datasets, i.e., a cross-sectional study for the characterization of foveal cystoid spaces and a longitudinal study, to evaluate the clinical relevance of OCT findings in eyes treated with anti-VEGF drugs. We retrospectively reviewed treatment-naïve center-involved DME accompanied with foveal cystoid spaces on which OCT images of sufficient quality were obtained. The inclusion criteria were 1. center-involved DME, 2. the presence of cystoid abnormalities at the foveal center, and 3. written informed consent. The exclusion criteria were as follows: 1. severe media opacity affecting visual function or image acquisition, 2. any other chorioretinal diseases, 3. any previous treatment for DME or macular diseases, 4. cataract surgery within 3 months, and 5. any intraocular surgery other than cataract surgery within 12 months. If both eyes met these criteria, we selected right eyes.

In another independent study, we retrospectively reviewed patients with center-involved DME who received IVR injections for 24 months or longer. The participants in the longitudinal study did not at all overlap with those in the cross-sectional study. The inclusion criteria at baseline were adults ≥ 20 years with diabetes mellitus, visual impairment due to center-involved DME, the presence of cystoid abnormalities at the foveal center, and written informed consent. The exclusion criteria at baseline were media opacity affecting VA, other chorioretinal diseases, angiogenic complications (neovascular glaucoma, vitreous hemorrhage, or tractional retinal detachment), any past intervention for DME (anti-VEGF therapy, focal/grid photocoagulation, vitreoretinal surgery, and intraocular or periocular corticosteroids), retinal photocoagulation within 6 months, intraocular surgery other than cataract extraction, and cataract surgery within the previous 3 months. We additionally excluded eyes that met the following criteria: 1. drop-out during 24-month follow-up due to patient’s inconvenience or desire to terminate treatment, 2. patient’s desire or doctor’s discretion to switch to other treatment during 12-month follow-up, and 3. patient’s desire or doctor’s discretion to switch to vitrectomy or intraocular or periocular corticosteroids.

All research and measurements were performed in accordance with the tenets of the Declaration of Helsinki and under the approval of the study protocol by the Kyoto University Graduate School and Faculty of Medicine, Ethics Committee. Written informed consent was obtained from all participants before study enrollment.

### OCT

Best-corrected decimal VA was measured and converted to logMAR VA. After comprehensive ophthalmic examination, SD-OCT images were obtained using Spectralis OCT (Heidelberg Engineering, Heidelberg, Germany) and the raster scan mode and cross-hair mode (30-degree) of the manufacturer’s software were applied as previously described^32^. Subsequently, two-dimensional maps were created, and the mean retinal thickness in the CSF was measured using the equipped software. We then determined center-involved DME according to the definition in a recent publication^33^.

In addition, we qualitatively evaluated the OCT findings of medium or large foveal cystoid spaces (≥250 μm in horizontal width, as defined in a previous publication^5^) in the central 1 mm of the retinal sections. We excluded smaller cysts, because the structural characterization is difficult in such cysts. We first determined the foveal center where inner retinal layers were absent and the central 1 mm areas in each OCT image. We evaluated the OCT findings, heterogeneous or homogenous reflectivity and hyperreflective foci in cystoid spaces within 1 mm in the vertical sectional images^9^. The height and width of each cystoid space were measured using the caliper of the manufacturer’s software. The OCT reflectivity was calculated in each foveal cystoid space as previously described^9^. Briefly, the margin of cystoid spaces was traced and the mean reflectivity level in the area encircled was quantified using image processing software (Photoshop, Adobe Systems, San Jose, CA). We compared it to the reflectivity levels of the vitreous cavity (as 0) and never fiber layer (NFL) (as 100) and calculated the relative reflectivity as arbitrary unit (AU) according to the formula:

$${\rm{Relative}}\,{\rm{reflectivity}}\,({\rm{AU}})={\rm{reflectivity}}\,({\rm{cystoid}}\,{\rm{space}})-{\rm{reflectivity}}({\rm{vitreous}})/{\rm{reflectivity}}({\rm{NFL}})-{\rm{reflectivity}}({\rm{vitreous}})\times 100$$

We defined a *hyperreflective wall in foveal cystoid spaces* as a novel OCT finding. We selected cystoid spaces with medium or large size (≥250 μm in horizontal width^5^) within central 1 mm areas on sectional OCT images. We first determined the smooth contour of round or oval-shaped cystoid spaces (Fig. 1). The walls of cystoid spaces are usually composed of surrounding parenchyma or thin lines with high reflectance due to the optical differences at the boundaries (Fig. 2B,C). ‘Plot profile’ function in ImageJ version 1.52 software (NIH, Bethesda, MD) demonstrated the differences in reflectivity levels between cystoid spaces and retinal parenchyma (right column in Fig. 2). Parts of cystoid spaces were sometimes outlined by thick bands (≥ 40μm) which had higher reflectivity levels than nearby parenchyma (Figs. 1D and 2A). This OCT finding was defined and referred to as *hyperreflective walls in foveal cystoid spaces* in this study, after the discrimination of hyperreflective deposits within round or oval-shaped cystoid spaces (Fig. 1C). Eyes with this OCT finding in either horizontal and/or vertical sectional images were defined as *eyes with hyperreflective walls in foveal cystoid spaces*.

We quantitatively evaluated the reflectivity levels and thicknesses of the hyperreflective walls. Using the caliper of the manufacturer’s software, we manually measured the thicknesses at three different places and calculated the mean thicknesses. The reflectivity of hyperreflective walls were quantified using Photoshop as in the case of that of cystoid spaces. We manually traced the margin of hyperreflective walls and measured the mean reflectivity in the areas encircled. The relative reflectivity levels were calculated according to the formula as described above.

We investigated whether parafoveal cystoid spaces were accompanied with hyperreflective walls in the cross-sectional study. Cystoid spaces between 0.5 and 1.5 mm from the foveal center were determined as parafoveal cystoid spaces on the vertical sectional images. We evaluated the hyperreflective walls in parafoveal cystoid spaces with 250 μm or more horizontal width, as in the case of walls in foveal cystoid spaces.

We further evaluated the DRIL and EZ disruption on the sectional OCT images as described previously^5,34^. Briefly, DRIL was determined as the horizontal extent where any boundaries between the ganglion cell layer-inner plexiform layer complex, inner nuclear layer, and outer plexiform layer were not identified^5^. The horizontal length of DRIL extent was measured using the caliper of the manufacturer’s software. The disrupted EZ line was defined as the horizontal length (%) of absent EZ line, after the exclusion of faint or intact EZ line^34^. Further, hyperreflective foci in the inner or outer retinal layers were defined as those between the inner limiting membrane and external limiting membrane (ELM) or those between the ELM and retinal pigment epithelium (RPE), as described previously^35^. Two independent retinal specialists evaluated qualitative and quantitative OCT findings.

Post-hoc analyses in clinical trials reported that reduced macular thickness was maintained in the second year despite the significant decrease in the treatment frequency of IVR injections^16,18^. We therefore investigated the remission of DME under PRN dosing. The absence of macular thickening at each time point does not necessarily mean the resolution or remission of DME, because we often observe the relapsing DME under PRN dosing. We speculated that cases without DME for 6 or more months may be determined as remission of DME. In the current study, eyes without center-involved DME from any time point (from 1-month to 18-month visit) to the 24-month visit were defined as those in *DME remission at that time point* as described previously^36^. The absence of cystoid abnormalities from any time point to 24-month visit was defined as *remission of cystoid abnormalities* in the similar manner.

### Intervention

IVR injections were performed according to a PRN regimen following three loading doses as described in the RESTORE study^16^. Ranibizumab (0.5 mg) was administered 3.5 mm posterior to the limbus after disinfection. As-needed injections at monthly visits were performed according to the retreatment criteria of the RESTORE study. At the 12-month visit and later, intravitreal aflibercept (IVA) injections, panretinal photocoagulation, or macular photocoagulation were also performed at the doctor’s discretion.

### Statistics

The results are shown as the median (interquartile range [IQR]). After testing the normal distribution using the Shapiro-Wilk test, parametric and nonparametric data were compared using Student’s t-test and the Mann-Whitney U-test, respectively. The differences in the sampling distributions were assessed using Fisher’s exact test. The Kappa coefficient or intraclass correlation coefficient (ICC) were employed to evaluate the concordance in the qualitative and quantitative factors, respectively. Cox proportional hazard model were applied to investigate whether DME remission was predicted by baseline factors. Multivariate analyses were employed in a stepwise forward manner. *P* < 0.05 was considered statistically significant. SPSS version 24.0 was used for statistical analyses (SPSS, Inc., Chicago, IL, USA).

## Supplementary information

Supplemental Material.
