# Supplementary material for: Hyperreflective Walls in Foveal Cystoid Spaces as a Biomarker of Diabetic Macular Edema Refractory to Anti-VEGF Treatment
Source: Sci Rep. 2020 Apr 29;10:7299. doi: 10.1038/s41598-020-64332-6 (PMC7190628; doi:10.1038/s41598-020-64332-6)
Supplement: Supplementary file 1 — Supplemental Material. [file 41598_2020_64332_MOESM1_ESM.pdf]

Supplementary Information

Hyperreflective Walls in Foveal Cystoid Spaces as a Biomarker of Diabetic Macular Edema Refractory to Anti-VEGF Treatment

Noriko Terada, MD, Tomoaki Murakami, MD, PhD, Akihito Uji, MD, PhD,

Yoko Dodo, MD, PhD, Yuki Mori, MD, Akitaka Tsujikawa, MD, PhD.

From the Department of Ophthalmology and Visual Sciences, Kyoto University Graduate School of Medicine, Kyoto, Japan.

Correspondence and requests for materials should be addressed to Tomoaki Murakami, MD, PhD, Department of Ophthalmology and Visual Sciences, Kyoto University Graduate School of Medicine, 54 Shogoin-Kawaracho, Sakyo, Kyoto 606-8507, Japan (phone: 81-75-751-3250; fax: 81-75-752-0933; email: mutomo@kuhp.kyoto-u.ac.jp)

E-mail: mutomo@kuhp.kyoto-u.ac.jp.

Table S1. Characteristics of patients in a cross-sectional study.

Parameter	
Eyes/patients	110/110
Age (years)	65 (60-72)
Men/women	69/41
Hemoglobin A1c (%)	7.0 (6.2-8.2)
Systemic hypertension (patients)	64
LogMAR VA	0.222 (0.064-0.523)
International classification	
Mild NPDR	2 eyes
Moderate NPDR	53 eyes
Severe NPDR	31 eyes
PDR	24 eyes
Pseudophakia	28 eyes
Panretinal photocoagulation	46 eyes
CSF thickness (μm)	437 (346-531)
Subretinal fluid	21 eyes (Kappa coefficient = 1.000)
Vitreoretinal abnormalities	15 eyes (Kappa coefficient = 0.960)
Disrupted EZ line (%)	3.3 (0.0-19.3) (ICC = 0.947)
DRIL (μm)	431 (235-569) (ICC = 0.919)
Hyperreflective foci in the inner retinal layers	87 eyes (Kappa coefficient = 0.890)
Hyperreflective foci in the outer retinal layers	40 eyes (Kappa coefficient = 0.902)

Table S2. Patient characteristics in a longitudinal study.

Parameter	
Eyes/patients	54/51
Age (years)	69 (63-74)
Men/women	33/18
Hemoglobin A1c (%)	7.1 (6.7-8.1)
Systemic hypertension (patients)	33
LogMAR VA	0.260 (0.155-0.523)
International classification	
Moderate NPDR	37 eyes
Severe NPDR	8 eyes
PDR	9 eyes
Pseudophakia	20 eyes
Panretinal photocoagulation	34 eyes
CSF thickness (μm)	455 (408-538)
Hyperreflective wall in foveal cystoid spaces	13 eyes (Kappa coefficient = 0.948)
Hyperreflective foci in foveal cystoid spaces	36 eyes (Kappa coefficient =0.919)
Subretinal fluid	6 eyes (Kappa coefficient = 1.000)
Vitreoretinal abnormalities	3 eyes (Kappa coefficient = 0.807)
Disrupted EZ line (%)	14.3 (0.0-31.6) (ICC = 0.962)
DRIL (μm)	647 (464-851) (ICC = 0.867)
Hyperreflective foci in the inner retinal layers	44 eyes (Kappa coefficient = 0.871)
Hyperreflective foci in the outer retinal layers	27 eyes (Kappa coefficient = 0.864)
IVR or IVA administrations<br><br>Additional treatment for DME<br>Additional treatment for DR	7 injections (5-10)<br>(*Switch to IVA in 7 eyes)<br>Focal photocoagulation 1 eye<br>Panretinal photocoagulation 1 eye

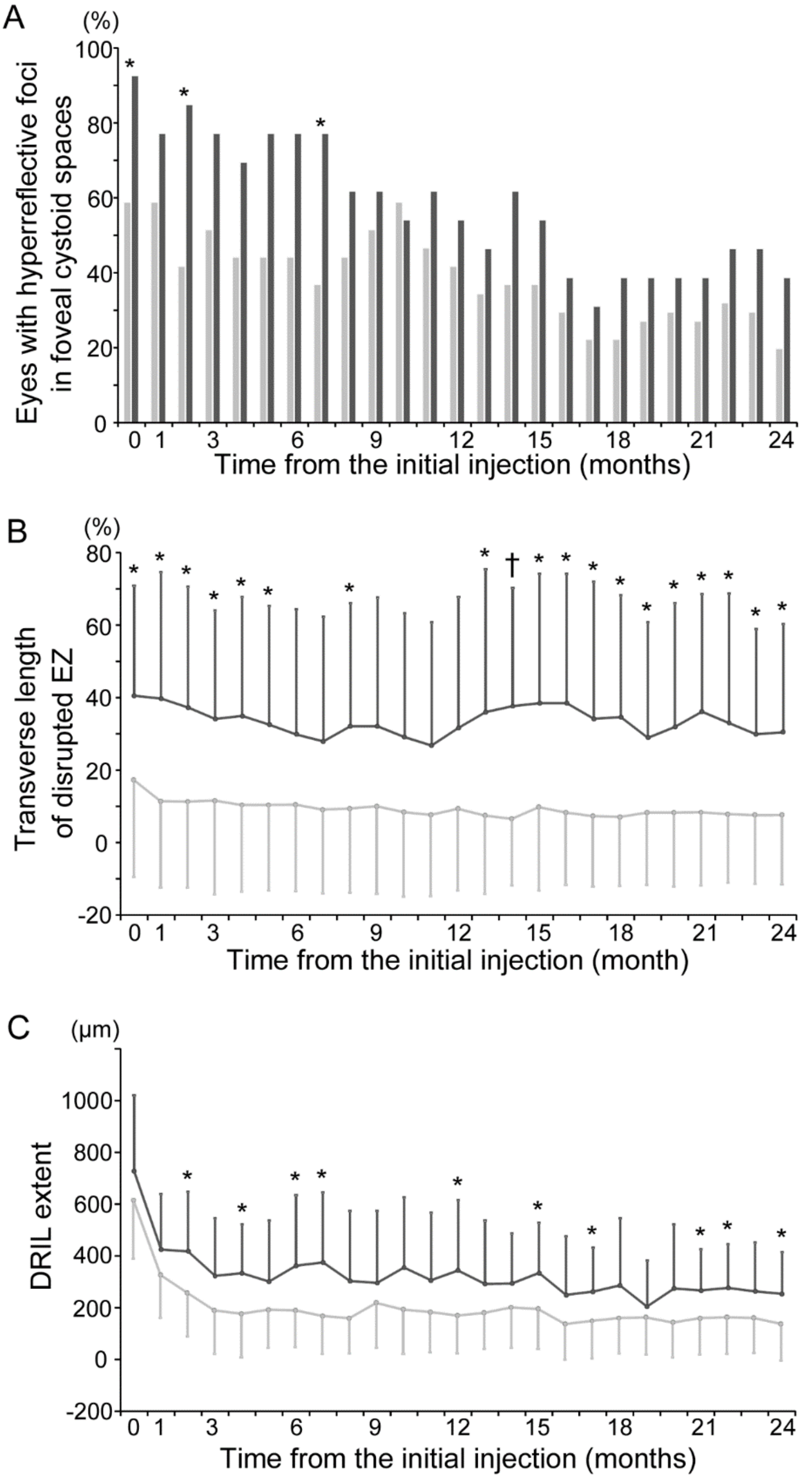

Figure S1. The courses of hyperreflective foci in foveal cystoid spaces, disrupted EZ line, and DRIL extent under as-needed ranibizumab injections.

Hyperreflective foci in foveal cystoid spaces (A), disrupted EZ line (B), and DRIL extent (C) in 13 eyes with hyperreflective walls in foveal cystoid spaces (dark gray) and 41 eyes without such findings (light gray). **P*<0.05; †*P*<0.01.
